# Diversification of the muscle proteome through alternative splicing

**DOI:** 10.1186/s13395-018-0152-3

**Published:** 2018-03-06

**Authors:** Kiran Nakka, Claudia Ghigna, Davide Gabellini, F. Jeffrey Dilworth

**Affiliations:** 1 Sprott Centre for Stem Cell Research, Regenerative Medicine Program, Ottawa Hospital Research Institute, Ottawa, ON K1H 8L6 Canada; 2 Istituto di Genetica Molecolare—Consiglio Nazionale delle Ricerche (IGM-CNR), Pavia, Italy; 3 Unit of Gene Expression and Muscular Dystrophy, Division of Genetics and Cell Biology, IRCCS San Raffaele Scientific Institute, DIBIT2, 5A3-44, via Olgettina 58, 20132 Milan, Italy; 4 Department of Cellular and Molecular Medicine, University of Ottawa, Ottawa, ON K1H 8M5 Canada; 5 Sprott Centre for Stem Cell Research, Ottawa Hospital Research Institute, 501 Smyth Rd, Mailbox 511, Ottawa, ON K1H 8L6 Canada

**Keywords:** Alternative splicing, Myogenesis, Muscle disorders, Muscular dystrophy, Co-transcriptional splicing, RNA-binding proteins, Proteome

## Abstract

**Background:**

Skeletal muscles express a highly specialized proteome that allows the metabolism of energy sources to mediate myofiber contraction. This muscle-specific proteome is partially derived through the muscle-specific transcription of a subset of genes. Surprisingly, RNA sequencing technologies have also revealed a significant role for muscle-specific alternative splicing in generating protein isoforms that give specialized function to the muscle proteome.

**Main body:**

In this review, we discuss the current knowledge with respect to the mechanisms that allow pre-mRNA transcripts to undergo muscle-specific alternative splicing while identifying some of the key *trans*-acting splicing factors essential to the process. The importance of specific splicing events to specialized muscle function is presented along with examples in which dysregulated splicing contributes to myopathies. Though there is now an appreciation that alternative splicing is a major contributor to proteome diversification, the emergence of improved “targeted” proteomic methodologies for detection of specific protein isoforms will soon allow us to better appreciate the extent to which alternative splicing modifies the activity of proteins (and their ability to interact with other proteins) in the skeletal muscle. In addition, we highlight a continued need to better explore the signaling pathways that contribute to the temporal control of *trans*-acting splicing factor activity to ensure specific protein isoforms are expressed in the proper cellular context.

**Conclusions:**

An understanding of the signal-dependent and signal-independent events driving muscle-specific alternative splicing has the potential to provide us with novel therapeutic strategies to treat different myopathies.

**Electronic supplementary material:**

The online version of this article (10.1186/s13395-018-0152-3) contains supplementary material, which is available to authorized users.

## Background

Sequencing of the human genome has identified ~ 20,000 protein-coding genes within its 3.3 billion base pairs of haploid DNA [1]. However, the functional proteome is an order of magnitude more complex due to the fact that cells are free to interpret these protein-coding genes in different ways. This alternate interpretation of the genome is permitted as most protein-coding genes exist as a series of exons separated by a non-coding intronic sequence that is removed from the precursor mRNA (pre-mRNA) through the process of splicing. During intron removal, the splicing machinery can decide to skip one or more exons from a single pre-mRNA transcript thus generating multiple mature mRNAs. This differential use of exons in the pre-mRNA is referred to as alternative splicing. In humans, the process of alternative splicing is highly prevalent, affecting 95% of multi-exonic protein-coding genes [2, 3] thus producing more than 80,000 distinct mRNAs (Current Gencode release). Intriguingly, a significant portion of these alternative splicing events displays cell, tissue, or condition-specific regulation [2, 3]. Thus, the diversity of exon usage in a pre-mRNA eventually determines its coding potential as well as its stability, contributing to the quantity as well as the quality of the proteins synthesized [4–7].

For a large fraction of transcripts, splicing of pre-mRNA occurs in concert with gene transcription [8, 9]. The coordination of the transcription and pre-mRNA maturation is facilitated by the RNA polymerase II (RNA Pol II), whose carboxy-terminal domain (CTD) interacts with proteins involved in 5′ capping, splicing, and 3′ end cleavage and polyadenylation [10]. These interactions with the RNA Pol II CTD allow for the coordinated association of splicing factors with their consensus binding sequences on the nascent transcript to recognize exon/intron boundaries for splicing to occur. The 5′ (or donor) and 3′ (or acceptor) sites of the exon/intron boundary are identified, respectively, by the consensus sequences AG|GURAGU and YAG|G (“R” for purine; “Y” for pyrimidines; “|” for splice site). In addition, a polypyrimidine tract of variable length and a regulatory element called the “branch point” with the consensus sequence “YNYURAY” (N for any nucleotide) are also found upstream of the 3′ splice site. Excellent reviews on the mechanisms of splicing have been published, and this area will not be covered in depth here [11–17].

**Fig. 1:**
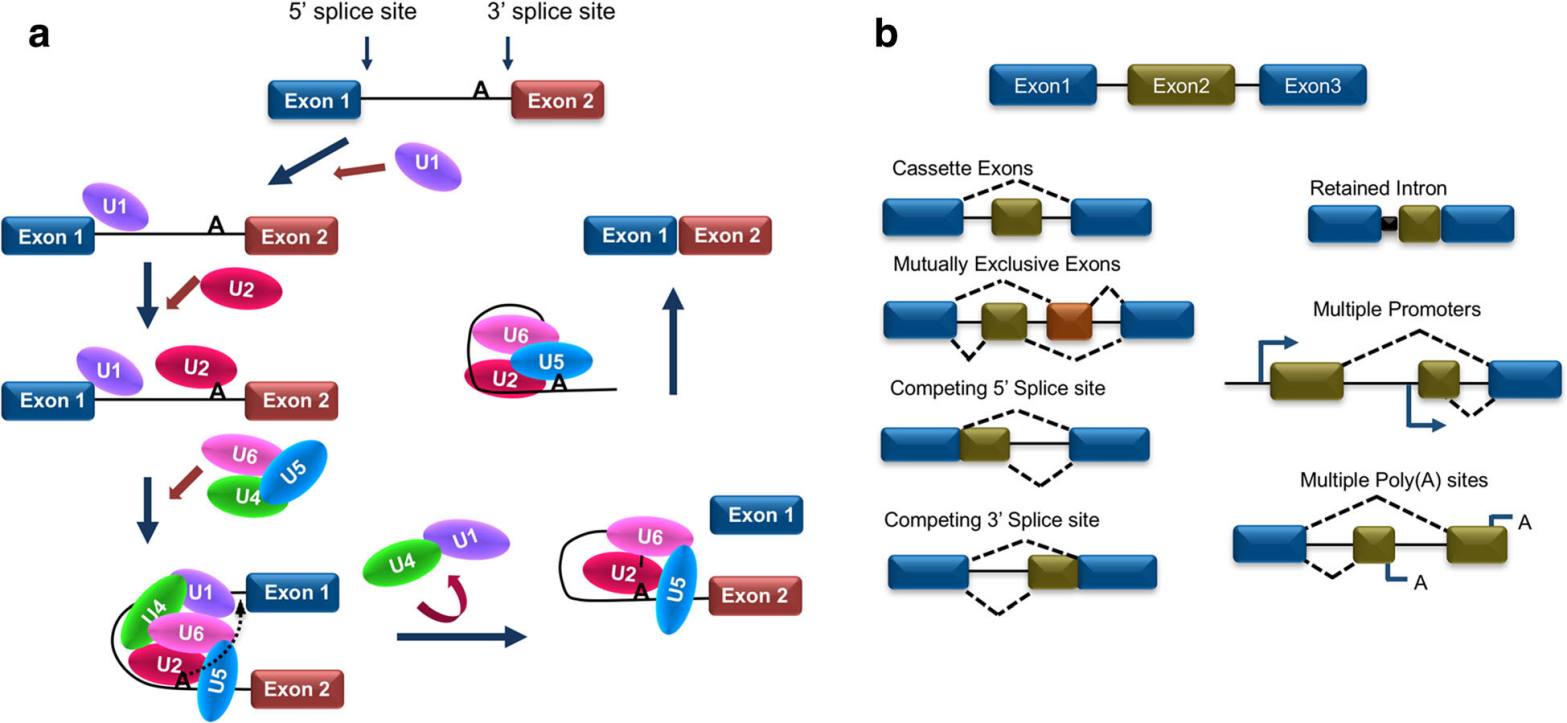
Mechanism and mode of pre-mRNA splicing. **a** The U1 snRNP particle recognizes the 5′ splice site while U2 recognizes the adenosine (A) of the branch point. Subsequently to the entry of the heterotrimeric (pre-assembled) U4/U6-U5 particle, several rearrangements lead to the catalytic activation of the spliceosome. This results in the displacement of U4 and U1 snRNPs with a concomitant attack of the 2′ OH of the adenosine of the branch site to the 5′ splice site generating a lariat intron-3′ exon intermediate. Finally, the 3′ OH of the free 5′ exon attacks the 3′ splice site, thus releasing the lariat intron and ligating the two exons. **b** Modes of alternative splicing reactions. Alternatively, spliced mRNAs are generated through inclusion (or skipping) of cassette exons, mutually exclusive exons, usage of 5′ and 3′ splice sites, intron retention, alternative promoters, or polyadenylation sites

Splicing of pre-mRNAs is mediated by a higher-order protein complex, termed as the spliceosome, which assembles splicing regulators at intron-exon junctions of pre-mRNAs [18–23] (Fig. 1). These regulators play essential roles during alternative splicing reactions where their ability to dictate exon definition (inclusion/exclusion) is controlled by several criteria including (i) the presence of specific *cis*-acting elements/sequences within the pre-mRNA that function as splicing enhancers or silencers, (ii) the RNA-binding proteins associated with the spliceosome that recognize the *cis*-regulatory elements in the pre-mRNA to stimulate or repress specific splicing events, (iii) long-range RNA-RNA interactions that use sequence complementarity in flanking introns to sequester specific exons for exclusion from the mature mRNA, and (iv) signal transduction pathways that target RNA-binding proteins thus indirectly altering their expression (or localization) or directly changing their activity through introduction of post-translational modifications (i.e., phosphorylation, acetylation, methylation, ubiquitination). When taken together, it can be surmised that the presence of a functional splicing regulator, which target local *cis*-regulatory elements, will be the strongest determinant as to whether an exon is excluded or included in the mature mRNA.

Alternative splicing can also be influenced through mechanisms that are extrinsic to the pre-mRNA sequence. Indeed, studies over the last decade have indicated that chromatin, including various chromatin modifiers and their associated histone modifications, plays a pivotal role in the regulation of alternative splicing [24–28]. Though the precise mechanism through which chromatin modifiers affect alternative splicing is not completely established, it is proposed that they function either by modulating the rate at which the RNA Pol II-associated splicing machinery travels across the transcribing gene or by directly orchestrating the recruitment of splicing regulatory factors [24–30]. To further complicate matters, DNA-binding transcription factors have recently been found as additional direct regulators of alternative splicing events [31]. While this is partially mediated by recruitment of splicing regulators to specific genes [32], evidence suggests that transcription factors can also bind RNA to modulate splicing activity [31].

From a mechanistic point of view, the spliceosomal machinery can generate multiple mature mRNAs by interpreting exon/intron boundaries in several distinct ways (Fig. 1b). These different interpretations, for the most part, fall into one of six subgroupings of alternative splicing events. The most common alternative splicing event is exon skipping. In this subgroup that constitutes 40% of the global splicing events in humans, an alternative exon is simply included or excluded (skipped) from the mature mRNA transcript [33]. Another prevalent subgroup is distinguished by the use of alternative splice sites within the exon. In this case, the alternative usage of distinct 3′ or 5′ splice sites constitutes 18.4 and 7.9% of human alternative splicing events, respectively [33]. A rarer alternative splicing event in humans is intron retention, in which an intron is retained in the mature mRNA [34, 35]. Finally, mutually exclusive exon incorporation is an important mode of alternative splicing in which only one of two alternative exons is incorporated into the mature mRNA at the expense of the adjacent alternate exon [36]. Distinct mRNAs from a single gene can also be generated through alternate promoter usage where the transcription of an upstream first exon causes a shift in downstream exon usage. Similarly, alternative mRNA polyadenylation generates transcripts with variant 3′ ends thus allows the cell to generate proteins with carboxy-terminal variants [37, 38].

Recent advances in the global analysis of alternative splicing by splicing-sensitive microarrays and mRNA-Seq together with the identification of direct targets of various RNA-binding proteins upon cross-linking immunoprecipitation (CLIP) have provided new insights into the contribution of different alternative splicing mechanisms to the establishment of tissue or developmental-specific gene expression programs [2, 39–44]. In particular, genome-wide analysis of RNA transcripts has revealed the importance of alternative splicing to the diversification of the muscle proteome. Here, we will discuss the current state of knowledge concerning the mechanisms controlling alternative exon usage in muscle, and how the factors that drive alternative splicing play a key role in ensuring the function of healthy musculature.

## Alternative splicing in the muscle

The skeletal muscle has an extraordinary potential to regenerate itself after muscle injury [45, 46]. This regeneration is mediated by a pool of quiescent adult stem cells called satellite cells (SCs), located along the basal lamina of myofibers [45, 46]. Upon activation by various growth factor signals, which are secreted from the damaged fibers and the responding immune cells, the Pax7-expressing SCs undergo a transition from a quiescent to an activated state by inducing myogenic factor 5 (MYF5) and myoblast determination protein (MYOD) [47–49]. Regulated conversion of SCs to mature myofibers requires a precise spatiotemporal expression of various proteins that determine SCs’ symmetric and asymmetric divisions, which eventually help in replenishing the SC pool as well as in regenerating the myofibers to form muscle [50]. The ability of PAX7, MYOD, and MYF5 to control SC fate transitions has been extensively studied and shown to be tightly regulated by transducers of extracellular signaling including the Wnt, Notch, TGFβ, and p38 MAPK pathways [51–54]. However, a largely overlooked contributor to the control of SC fate during muscle regeneration is the post-transcriptional gene regulatory networks, more specifically, alternative splicing events that often affect the proteomic diversity as well as their temporal expression. For better understanding the precision of gene regulation during differentiation, we offer a brief overview of both RNA-binding proteins and alternative splicing events that contribute to the formation and maintenance of healthy muscle. Moreover, we also focus on the impact of alternatively spliced variants in muscle disorders and their manipulation for therapeutic purposes.

The use of splicing-sensitive microarrays or RNA-Seq has allowed a systematic compendium of human transcript isoforms across multiple tissues highlighting that the skeletal muscle is among the tissues showing the highest number of tissue-specific splicing events [40, 55]. Studies by Barash et al. [41], on multi-exon containing pre-mRNA transcripts from 27 different mouse tissue types, indicated that among the studied 3665 cassette-alternate exons, 23% uniquely exhibit exon inclusion or exclusion in a muscle cell-specific manner [41]. Another study by Trapnell et al. [56], using high-throughput analysis of paired-end mRNA sequencing data obtained during different time points of skeletal myogenesis, revealed 12,712 previously unidentified variants of known genes expressed in differentiating myoblasts. Among these newly identified isoforms, 7395 (58%) harbor novel splice junctions while the rest of the variants have novel combinations of previously known splicing outcomes. In addition, these new mRNA isoforms are present either during the entire course of myogenic differentiation (581 isoforms) or during specific time points (3724 isoforms) of differentiation. An analysis of the transcript dynamics for these novel isoforms indicated that alternate promoter usage and differential splicing, rather than transcriptional changes, are the major contributors to the generation of these variant transcripts [56]. Indeed, transcript analysis across multiple time points identified 273 genes that give rise to novel isoforms by alternative splicing, while 70 genes undergo both transcriptional and splicing regulation during myogenesis [56]. The *Mef2* family genes *Mef2C* [57–61] and *Mef2D* [62–66], for instance, undergo both transcriptional and splicing regulation, with the latter being a predominant regulatory event indispensable for myogenesis [64]. Despite using the first generation splice-sensitive arrays, a study by Bland et al. [67] identified 95 alternative splicing events that undergo robust and conserved splicing changes during the course of myogenic differentiation. These studies highlighted that most splicing events during myogenesis involve alternative usage of cassette exons (86%), while a smaller fraction of the transcripts exhibits mutually exclusive exons (7%) or exhibits either a 5′ splice site (5%) or a 3′ splice site (2%) usage. We note that some of these values were generated using older, less precise microarray technology. Thus, it will be important to revisit the proportional distribution of these different splicing events in the muscle using RNA-sequencing data.

## Regulation of muscle-specific alternative splicing

**Fig. 2:**
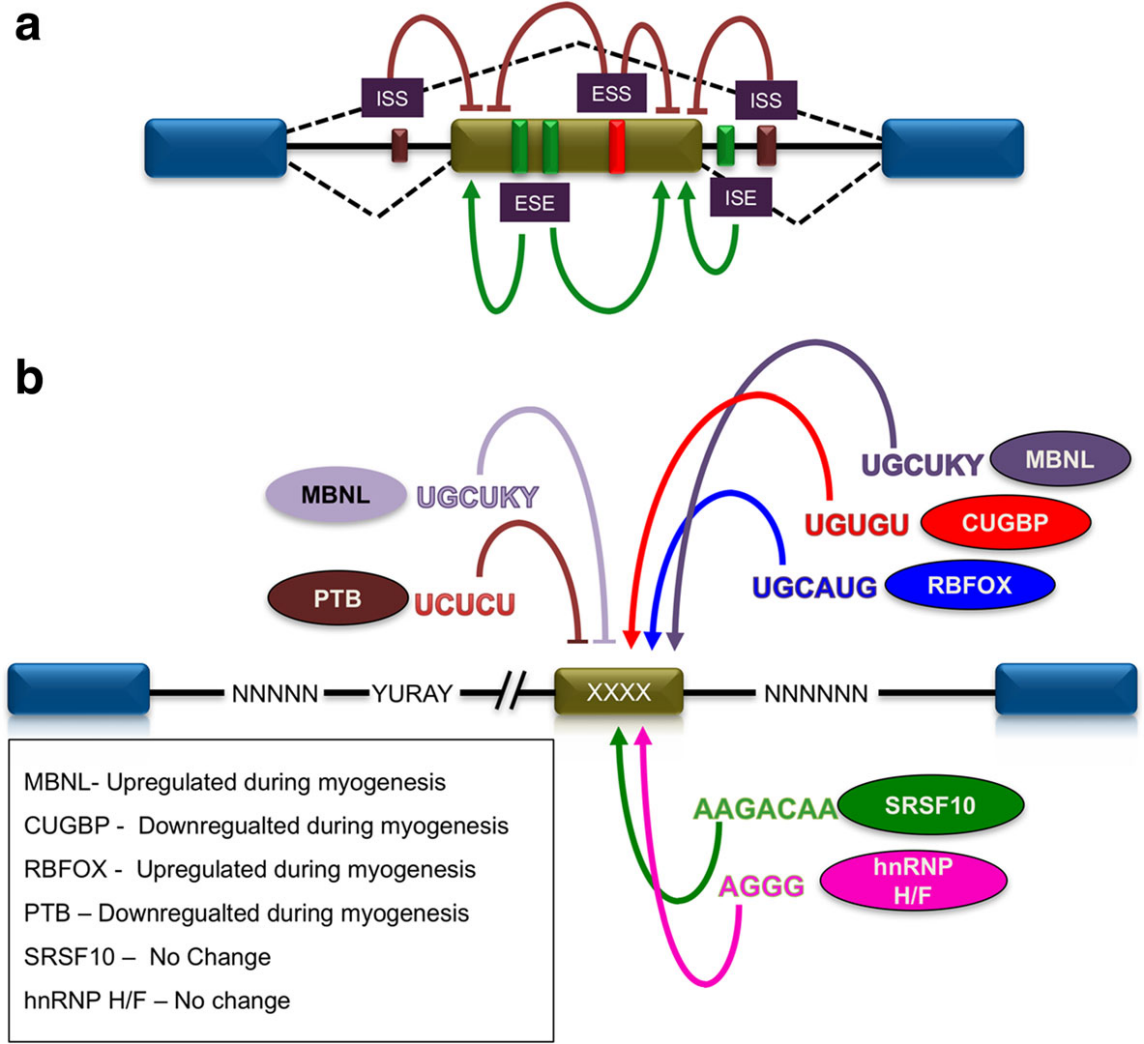
*Cis*-elements and *trans*-acting factors regulate pre-mRNA splicing during myogenesis. **a** Pre-mRNA harboring various *cis*-acting regulatory sequences able to promote (+) or inhibit (−) recognition of alternative splicing exons. Based on their position, these elements are called intronic splice silencers (ISS) and enhancers (ISE); exonic splice silencers (ESS) and enhancers (ESE). **b** A depiction of the enrichment of consensus *cis*-elements bound by various *trans*-acting factors acting in a coordinated manner to regulate alternative splicing of multiple transcripts during myogenesis. The upstream or downstream binding of the *trans*-acting factors relative to the alternative exon dictates its inclusion or exclusion. Protein mentioned in the box are some of the trans-acting factors that regulate muscle-specific alternative splicing and are differentially expressed during the course of myogenesis

In addition to the 5′ and 3′ splice sites, the generation of tissue-specific splice variants is largely determined by the presence of auxiliary *cis*-regulatory elements on the pre-mRNAs (Fig. 2a), which can be bound by *trans*-acting factors to either promote or inhibit exon recognition in a tissue-specific manner. For understanding the *cis*-regulatory elements (or splicing code) that modulate splicing decisions in a tissue-specific manner, the enriched RNA sequence motifs that are common within the exonic and/or intronic regions located around the alternative spliced exons were identified [68, 69]. Motifs identified as being enriched at alternatively spliced exons were characterized and classified as potential regulatory elements that are bound by specific *trans*-acting factors (Fig. 2b). Castle et al. [40] characterized eight regions (neighborhoods) in or adjacent to muscle-specific exons. Analysis of each neighborhood within 200 nucleotides of intronic region and 39 nucleotides of exonic region revealed the enrichment of a *cis*-element “UCUCU,” corresponding to the binding site of the *trans*-acting factor polypyrimidine tract-binding protein (PTB also called PTBP1 or hnRNP I), as the most abundant penta-nucleotide repeat found in intronic regions upstream of alternative cassette exons preferentially included in the skeletal muscle [40, 41, 70, 71]. Other motifs, such as “UGCAUG,” bound by members of the RNA-binding Fox protein family (RBFOX1 and RBFOX2) [72, 73]; “UGUGUG”, bound by CELF (CUG-BP1 and ETR-3 like factors) family proteins [73, 74]; and “UGCU,” bound by MBNL (muscleblind-like) proteins were shown to be enriched 10 to 80 nucleotides in the intronic regions downstream of the included cassette exons [40, 75, 76]. The “CUAAC” motif, which resembles the sequence bound by branch point binding protein BBP/SF1, is also reported to be enriched downstream of the included exons [77]. In contrast, no specific sequence elements were identified to be associated with muscle cell-specific skipped exons. This reflects the fact that exon skipping events are not as abundant during the course of myogenesis [78]. Together, these studies highlight the important role for unique *cis*-elements present in the intronic regions downstream of “to be included” exons as an important mediator of alternative splicing events that contribute to muscle-specific gene expression.

The differential exon usage linked to the occurrence of enriched “sequence motifs” is dependent on the expression (or regulated activity) of *trans*-acting factors that bind to these conserved sequences. Supporting this notion, a strong correlation has been observed between the temporal changes in expression of RNA binding *trans*-acting factors (such as RBFOX, CELF, MBNL, and PTB family members) and the timing of associated alternative splicing events in muscle differentiation [65, 66, 79, 80]. Demonstrating the need for specific splicing factors at distinct temporal stages of muscle homeostasis, the muscle-specific knockout of RBFOX1 did not affect satellite cell-mediated regeneration but did disrupt calcium handling and maximal force generation in adult muscle [81]. These phenotypic observations are consistent with the fact that RBFOX1 is upregulated during myogenic differentiation leading to the enhanced inclusion of alternative exons in transcripts encoding for MEF2D, Nebulin-related anchoring protein (Nrap), and myosin-binding protein-C (Mybpc1) [66, 81]. However, it should be noted that RBFOX1 can also direct the exclusion of exons such as in the case of the human mitochondrial ATP synthase γ-subunit where exon 9 is removed exclusively in the skeletal muscle cells [72, 82]. The decision as to whether RBFOX1 will include or exclude an exon depends upon the positioning of the UGCAUG motif relative to the alternatively spliced exon. Indeed, RBFOX1 is able to promote exon skipping when bound to UGCAUG motifs upstream the alternative exon, whereas it promotes exon inclusion when bound to motifs located downstream of the alternative exon [72, 83]. Similarly, the *trans*-acting factors CELF, MBLN1, and PTB can act as splicing activators (exon inclusion) or repressors (exon skipping) depending on the positioning of their cognate motif relative to the alternatively spliced exon [84, 85]. Similar to RBFOX1, MBLN1 is upregulated during differentiation, allowing inclusion of the β exon into the *Mef2D* transcripts [86]. The consequence of this β exon inclusion in MEF2D is not known. In contrast, PTB protein levels decrease during differentiation [79] allowing Capzb transcripts to incorporate exon 9 [87]. It should be noted that many splicing events are regulated by the combined activity of multiple *trans*-acting splicing factors. In the case of *α-actinin* pre-mRNA, RBFOX1 works in consort with the CELF protein Brul (Bruno-like) by antagonizing the effects of PTB to enhance the inclusion of mutually exclusive muscle-specific exon SM, generating a muscle-specific isoform of the protein [72, 84, 85]. In another example, MBNL1 functions with PTB to regulate splicing of *α-tropomyosin* [88]. In contrast, CELF1 functions antagonistically with MBLN1 to control fetal to adult splicing transitions [89]. In this context, reduction in CELF1 protein levels allow MBLN1 to establish splicing of transcripts to generate the adult isoforms for *calcium voltage-gated channel subunit alpha subunit-1*(*CACNA1S*), *cardiac troponin T* (*cTNT*), and *INSR* in the cardiac and skeletal muscle [89, 90]. Analogously, downregulation of the hnRNPs H/F-associated proteins DDX5 and DDX17 during terminal muscle differentiation facilitates the inclusion of weak alternative exons wherein G-tracts or G-quadruplex structures performing a splicing-enhancer function [91]. In addition, the spatial distribution of various splicing factors including CELF, MBNL, etc., also regulates alternative splicing events, especially during myotonic dystrophy (DM) pathogenesis [85, 92–95]. Enhanced nuclear localization of CELF and sequestration of MBNL proteins as distinct foci in DM cells but not in non-DM cells along with perturbed splicing events in DM pathogenesis underscores CELF/MBNL-dependent misregulated splicing during myotonic dystrophy [92, 94, 96]. Thus, the spatiotemporal expression of key splicing factors plays an essential role in establishing muscle-specific splicing.

The ability of a splicing factor to mediate exon inclusion/exclusion can also be modulated through post-translational control of its activity. SRSF10 provides an example of how post-translational modifications can regulate the activity of a *trans*-acting splicing regulatory factor as studies have shown that the phosphorylation status of the protein dictates whether it is an activator (phosphorylated) or repressor (unphosphorylated) of alternative splicing [97]. Though the regulation of SRSF10 function has not been studied in the context of the muscle, knockout of the splicing factor during development results in defects in myofiber formation [98]. Characterization of C2C12 cells expressing reduced levels of SRSF10 showed that it regulates alternative splicing of numerous transcripts including *FMR1 Autosomal homolog 1* (*Fxr1*) exons 15 and 16, *leucine-rich repeat interacting protein 1* (*Lrrfip1*) exons 16 and 17, *F-actin-capping protein subunit beta* (*Capzb*) exon 9, and *Mef2A* exon 9 during myogenesis [98].

Adding complexity to the splicing process, pre-mRNAs encoding for splicing factors themselves can undergo alternative exon usage, which can then influence downstream muscle cell type-specific alternative splicing, as illustrated by RBM4-mediated induction of a *PTB* isoform lacking exon 11 during myogenesis [99]. The skipping of exon 11 is detrimental to the *PTB* transcript as it introduces a premature termination codon in the mRNA thus activating the nonsense-mediated mRNA decay pathway. As PTB directly regulates its own splicing, an autoregulatory loop is established that controls the levels of PTB in the cell and thus the extent to which transcripts are alternatively spliced [99]. This mode of autoregulated splicing is also observed in the muscle cells for *Mbnl1* transcripts. MBNL1 promotes skipping of its own exon 5, which encodes an 18 amino acid fragment important for MBLN1 subcellular localization, RNA binding affinity, and alternative splicing activity [100, 101].

## Role for alternatively spliced isoforms in muscle

The functional analysis of distinct proteins generated by alternative splicing is an underdeveloped area of muscle biology. However, there are multiple examples that highlight the importance of alternatively spliced isoforms to proper muscle development and function (Additional file 1: Table S1). The following are a few salient examples to illustrate how alternative splicing regulates myogenesis, muscle contraction, and calcium handling in myofibers.

### Transcription factors: alternative splicing of Mef2 family genes

**Fig. 3:**
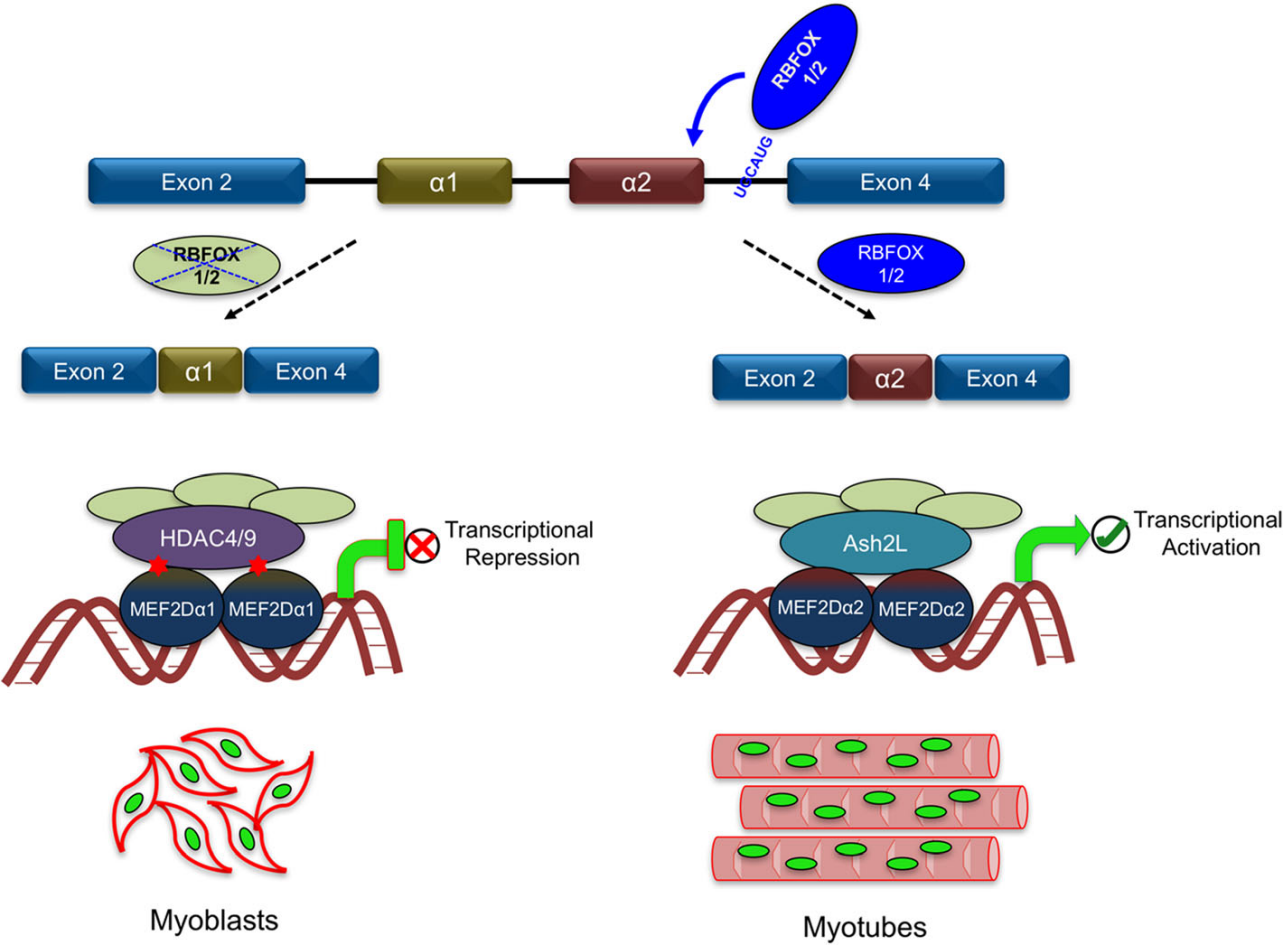
Tissue-specific regulation of pre-mRNA splicing. During myogenesis, induced expression of RBFOX1 and RBFOX2, which binds to the consensus downstream intronic element, promotes the production of the *Mef2Dα2* alternative spliced variant. MEF2Dα2 facilitates the expression of late differentiation genes to support the formation of myotubes

During myogenesis, the splicing regulation of the MEF2 family of transcription factors plays a key role in determining the timing of gene activation required for the formation of multinucleated myotubes [102]. In the muscle, the MEF2 family members MEF2A, MEF2C, and MEF2D are well known to act as transducers of various cell signaling pathways thus controlling gene activation or repression in response to post-translational modifications to modulate myogenesis [103, 104]. In the case of MEF2D, a muscle-specific transcript is generated by the inclusion of exon α2 at the expense of the mutually exclusive exon α1 to form the *Mef2Dα2* isoform [64]. MEF2Dα1 and MEF2Dα2 are both expressed in the muscle, where MEF2Dα1 acts as a transcriptional repressor, while MEF2Dα2 acts as a transcriptional activator. The antagonizing functions of these two MEF2D isoforms result from the fact that the α1 exon encodes a docking site for the kinase PKA that results in phosphorylation of MEF2Dα1 (at S119 and S190) but not MEF2Dα2 [64]. The phosphorylated MEF2Dα1 protein forms an inhibitory complex along with HDAC4 and HDAC9 to repress gene expression [105, 106] (Fig. 3). Exchange of the α1 exon with the α2 exon generates an isoform that evades the HDAC inhibitory complex, allowing MEF2Dα2 to function as a transcriptional activator. Temporal analysis of splicing shows that generation of the transcriptionally active MEF2Dα2 isoform occurs in the late stages of terminal muscle differentiation, at a time point that coincides with the upregulation of RBFOX1 and RBFOX2 [66]. At the molecular level, several studies revealed that RBFOX1/2 regulate *MEF2D* alternative splicing through binding to a “UGCAUG” motif located downstream of the α2 exon thus promoting inclusion of this muscle-specific exon (Fig. 3) [65, 66]. Like *Mef2D*, the *Mef2C* gene also encodes a muscle-specific isoform generated through the mutually exclusive usage of exons α1 and α2—*Mef2Cα1* and *Mef2Cα2*. Again, the MEF2Cα1 isoform strongly associates with repressor proteins like HDAC4 or HDAC5 to inhibit muscle-specific gene expression, while MEF2Cα2 acts as a transcriptional activator to help drive MyoD-dependent gene expression [61]. Intriguingly, the activation of the kinase SRPK3, which specifically phosphorylates the SR family of splicing factors, regulates the mutually exclusive splicing of *Mef2Cα1* and *Mef2Cα2*, promoting α2 exon inclusion to drive differentiation [61]. However, during differentiation, MEF2Cα1 continues to be expressed, though at a reduced rate, playing a key role in the activation of the AKT-mTOR pathway [107]. A less well-characterized isoform of the *Mef2C* gene is generated by alternative usage of the γ exon (MEF2Cα1γ), where the γ domain also harbors phosphorylation sites that control the repressive activity of the transcription factor to provide an additional level of regulation [59, 108]. Thus, alternative splicing of *Mef2D* or *Mef2C* plays a key role in determining whether these transcription factors will act as transcriptional activators or transcriptional repressors, a decision that will have opposing outcomes on Mef2-dependent gene expression. There are no known splicing isoforms of the myogenic regulatory factors (MYOD, MYF5, Myogenin, and MRF4) as each protein is encoded by gene structures containing only three exons. However, the satellite cell transcription factors PAX7 and PAX3 do undergo alternative splicing [109, 110]. Among the splice variants produced by both *Pax7* and *Pax3*, there is an isoform with an additional glutamine residue in the linker region separating the two DNA binding domains, allowing for altered binding affinity [110]. The functional consequence of these *Pax3* and *Pax7* splicing events on muscle formation and/or function has yet to be established.

### Alternative splicing of genes encoding for contractile proteins

**Fig. 4:**
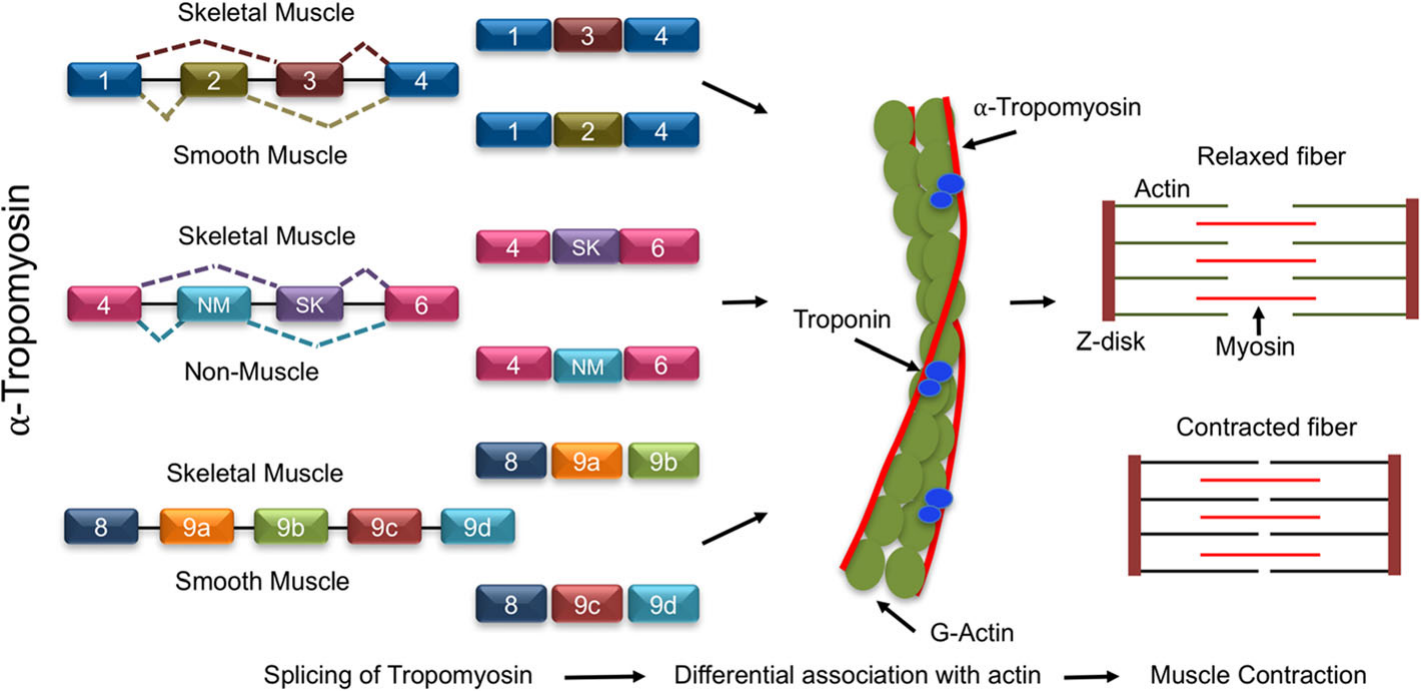
Alternative splicing of contractile protein gene *tropomyosin*. *Tropomyosin* undergoes cell type-specific alternative splicing and regulates muscle contraction. Exons 2 and 3 are mutually exclusive, and transcript with exon 3 is skeletal muscle specific. Also, the inclusion of SM exon is skeletal muscle specific, while NM exon is included in non-muscle cells. The inclusion of exons 9a and 9b of *tropomyosin* transcript is unique for isoforms of the skeletal muscle. Cell type-specific inclusion of exons alters the affinity with and polymerization of actin filaments, contributing to altered muscle contraction

In the muscle cells, alternative splicing plays an important role in controlling the functional diversity of contractile proteins [111], by generating different types of sarcomeres that exhibit divergent physiological properties. Among the genes encoding for the many contractile proteins, *tropomyosin* (*TM*) and *troponin* (*TnT*) are well-studied paradigms used to understand muscle cell-specific alternative splicing as they contain multiple alternative cassette exons that are either included, skipped, and/or used in a mutually exclusive manner [112, 113]. *TM* is expressed in all cell types [111, 114] and generates tissue-specific splice variants by making use of mutually exclusive and alternative exons, alternative promoters, and polyadenylation sites [111, 114] (Fig. 4). For instance, exon 3 of *α-TM* gene is preferentially included in all cell types except smooth muscle wherein exon 2 is incorporated to generate a smooth muscle cell-specific *α-TM* variant [113]. In humans, the *α-TM* gene has two versions of exon 5 that are used in a mutually exclusive manner to generate the *α-TM*_*NM*_ or *α-TM*_*SK*_ variants [115]. Expression of the *α-TM*_*SK*_ variant is exclusive to skeletal muscle, whereas *α-TM*_*NM*_ is the ubiquitous *α-TM* mRNA found in non-muscle cells [116] (Fig. 4). The inclusion of the SK-specific exon is an active process requiring a splicing machinery restricted to the muscle cells as the genetic deletion of the *NM* exon was not sufficient to have the *SK* exon incorporated into the *α-TM* mRNA in non-muscle cells [116]. Recent studies using biochemical purification to functionally characterize different tissue-specific isoforms of *α-TM* showed the muscle-specific isoform *α*-*TM*_*SK*_, which harbors exon 9a (as well as 1a, 2b), displays a higher relative affinity for F-actin filaments compared to isoforms that did not contain exon 9a, altering the kinetics of actin polymerization [117, 118]. The importance of alternative splicing in generating muscle-specific isoforms is also exemplified by the *β-TM* gene. In the muscle, *β-TM* transcripts incorporate the mutually exclusive exons 7 and 10, while transcripts in fibroblasts incorporate exons 6 and 11 (at the expense of exons 7 and 10) to encode for *TM-1* [119]. The stringent restriction of exons 7 and 10 usages to muscle is attributed to the binding of *trans*-acting factors to the *cis*-regulatory elements on the pre-mRNA that actively inhibit their incorporation into transcripts in non-muscle cells [115, 116]. In the case of *TnT*, the muscle cell-specific isoforms *TnT*_*F*_ (fast skeletal), *TnT*_*S*_ (slow skeletal), and *TnT*_*C*_ (cardiac muscle) are generated from a single gene in which transcripts include either exon 16 or exon 17 in a mutually exclusive manner and vary in their inclusion/skipping of exons 4 through 8 [120]. Similarly, the mutually exclusive use of either exon 2a or 2b distinguishes the smooth muscle isoform from the striated/skeletal muscle isoform of *α-tropomyosin* [113, 121]. These muscle-specific splicing events have been found to be regulated by the differential expression of certain *trans*-acting factors such as RBM4, PTB [122, 123], and CUGBP1 [124] that facilitate the inclusion or skipping of mutually exclusive exons, while the default splicing pattern is produced by general splicing machinery [111]. In addition to these tissue-specific *trans*-acting factors, the strength of the polypyrimidine tract as well as the branch point sequence also determines the inclusion or exclusion of mutually exclusive exons [111, 115, 116]. Functionally, these tissue-specific splice variants of the skeletal muscle are important for altering the calcium handling of the contractile apparatus via their association with Ca^2+^ regulatory proteins and thus play key roles in determining the kinetics of muscle contraction within muscle fibers [125, 126].

### Functional significance of alternative splicing in muscle cell mitochondria

The skeletal muscle displays a striking ability to maintain a moderate energy consumption during long-term low-intensity contractions while being able to rapidly increase energy consumption when explosive contractions are needed [127]. Ca^2+^ uptake plays a key role in maintaining energy homeostasis in response to the different contracting needs of the skeletal muscle [128–130]. Recent studies looking at changes in gene expression during post-natal development showed that calcium handling genes do not change their expression levels but instead undergo splicing changes that allow a transition from embryonic to adult protein isoforms [44]. An interesting example is provided by MICU1, one of the components of the mitochondrial calcium uniporter (MCU), the highly selective channel responsible for Ca^2+^ entry through the ion-impermeable inner mitochondrial membrane [131]. Muscle-specific alternative splicing generates the *MICU1.1* variant that is characterized by the inclusion of a highly conserved micro-exon encoding four amino acids that are sufficient to dramatically alter the properties of the MCU. Heterodimerization of the muscle-specific MICU1.1 with MICU2 in the mitochondria generates a variant complex with a greatly increased binding affinity for calcium, effectively lowering the [Ca^2^^+^] threshold required to sustain mitochondrial calcium levels needed for ATP synthesis during muscle contraction [132].

### Alternative splicing of genes involved in excitation-contraction coupling

**Fig. 5:**
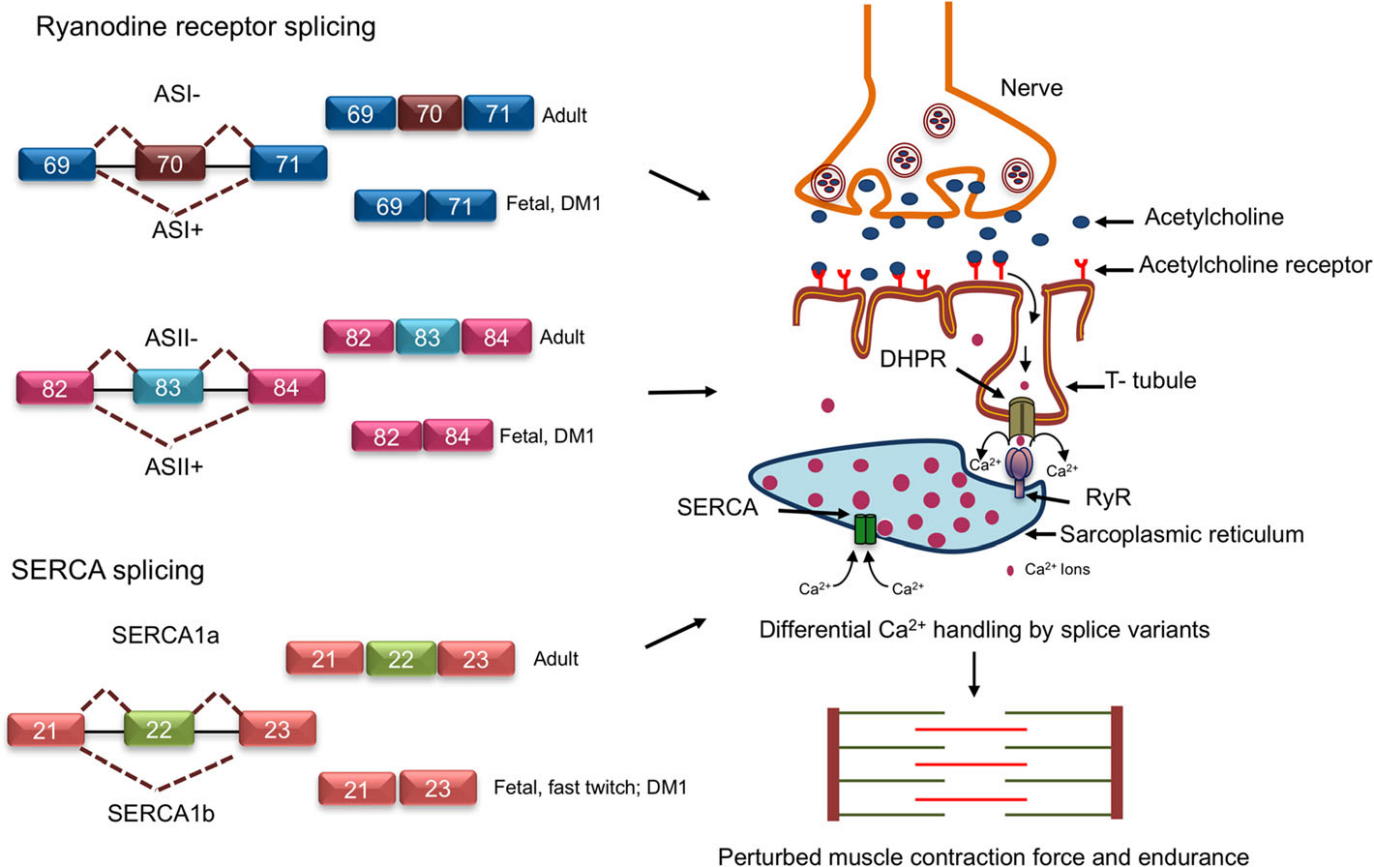
Alternative splicing of genes involved in excitation-contraction coupling. Acetylcholine secreted from the synaptic nerve ends binds to acetylcholine receptors and generates an action potential that transmits through T-tubule, activating ryanodine receptors to efflux Ca^2+^ from sarcoplasmic reticulum and SERCA channels facilitate influx of Ca^2+^. *Ryanodine receptor* and *SERCA* channel transcripts undergo “fetal-to-adult” temporal shift in alternative splicing at exons 29 and 33 and exon 22, respectively. Fetal splice variants of *RyR1* and *SERCA* (also observed in DM pathogenesis) are less efficient in ATPase and Ca^2+^ handling

Skeletal muscle contraction and relaxation is regulated by rapid and transient changes in the concentration of free Ca^2+^ in the cytoplasm of the muscle fiber (myoplasm). The excitation-contraction coupling determines the force of muscle contraction [133]. In a hierarchal order of events, excitation-contraction coupling (ECC) is initiated with an action potential on the plasma membrane that is spread through the transverse T-tubule system. This potential is detected by dihydropyridine receptors (DHPR, L-type Ca^2+^ channel Ca_V_1.1) leading to association with sarcoplasmic reticulum (SR) ryanodine receptors (RyR), which release Ca^2+^ into the myoplasm [134]. This is followed by a transient activation of the Ca^2+^ buffering system and muscle contraction followed by a rapid movement of Ca^2+^ back to the SR via the SR Ca^2+^ adenosine triphosphatase (SERCA) [134] (Fig. 5). Aberrant splicing of the pre-mRNAs encoding for calcium channels involved in Ca^2+^ homeostasis such as *Ca*_*V*_*1.1*, *Ca*_*V*_*1.2*, *RyR*, and *SERCA* is often observed in muscular dystrophy [135–138]. In the case of *Ca*_*V*_*1.1* (*CACNA1S*) alternative splicing of exon 29 generates a calcium conducting Ca_V_1.1e that enhances calcium influx and generates spontaneous calcium sparklets in muscle fiber during EC coupling, resulting in reduced force and enhanced endurance as observed in myotonic dystrophies DM1 and DM2 [138, 139]. Similarly, in the vascular smooth muscle and heart muscle cells, *Ca*_*V*_*1.2* is alternatively spliced by RBFOX2 generating a variant with exon 9 inclusion (Ca_v_1.2SM) and exon 33 (Ca_v_1.2CM) skipping during hypertension [140]. Functionally, these *Ca*_*v*_*1.2* splice variants differ in their Ca^2+^ window current, where the Ca_v_1.2SM isoform can stimulate Ca^2+^ influx at a much-reduced action potential for basal contractility during blood flow [141]. In addition, the peptide region encoded by exon 9 in *Ca*_*V*_*1.2* is found to be sensitive to calcium channel blockers [142] and harbors a potential protein kinase A phosphorylation site. However, it remains to be understood how phosphorylation status on this site affects the sensitivity of Ca_V_1.2 calcium channel [143, 144]. Ryanodine receptors are a specific class of intracellular calcium channels encoded by different genes in various tissues [145]. In the skeletal muscle, these are encoded by the *RyR1* gene, while the heart and smooth muscles express the *RyR2* gene. Skipping of exon 70 (ASI) that generates RyR1-ASI(−) is predominant in embryonic skeletal muscle while ASI(+) is predominant in adult skeletal muscle [146]. Intriguingly, RyR1 ASI(−) is a less active and less stably opened channel compared to ASI(+) and exhibits a significantly lower incidence of Ca^2+^ oscillations, with ASI(+) myotubes hold higher resting Ca^2+^ [146, 147]. Alternative splicing of exon 83 generates ASII(−) and ASII(+) variants. However, the temporal switch in splicing events for ASII occur much earlier (post-natal day 8) than for ASI (post-natal day 16) [146, 148] (Fig. 5). These temporal shifts in splicing in a tissue-specific manner illustrate the functional diversity [148] and specificity of muscle-specific alternative splicing. This specificity can be further illustrated with *SERCA1* (*sarco/endoplasmic reticulum Ca*^*2+*^*-ATPase 1*) alternative splicing where exon 22 is differentially spliced and generates *SERCA1a* with exon 22 included, which is predominant in adult fast-twitch muscle [149]. Exon 22 is skipped in neo-natal fast-twitch muscle fiber to encode for *SERCA1b* splice variant. In addition, SERCA1b is abundantly expressed in fast twitch fibers in DM1 pathogenesis. Despite SERCA1a and SERCA1b having similar affinity for both ATP and Ca^2+^, SERCA1b has lower ATPase and Ca^2+^ handling activity [150] (Fig. 5). Splicing in the *SERCA2* gene encodes for cell type-specific splice variants [146, 151, 152]. *SERCA2a*, wherein exon 21 is skipped, is expressed in the slow-twitch skeletal muscle fibers and cardiac muscles. *SERCA2d*, another skeletal muscle-specific isoform, retains intron 19 [146], and the expression of this isoform is downregulated in DM1 muscle tissue [153]. Albeit SERCA2a is demonstrated to regulate contractile calcium cycling, the distinct regulatory roles of SERCA2 splice variants is unclear [154]. Altogether, the above examples illustrate the functional significance of the divergent proteome that is generated upon tissue-specific alternative splicing. Nevertheless, given the extent of alternative splicing events identified in differentiating and mature muscle, there remains much to be learned about the role of alternative splicing variants in ensuring healthy muscle function as well as the molecular mechanisms responsible for their production.

## Alternative splicing and muscle disorders

Given the importance of splicing to ensure the highly specialized function of the skeletal muscle, it is not surprising that mutations that disrupt the alternate exon selection contribute to a large number of myopathies. Among the different myopathies, the mechanism through which splicing becomes altered can vary, but include (1) mutations in *cis*-regulatory elements that prevent the binding of *trans*-acting factors, (2) by altering expression of key *trans-*acting factors, or by (3) sequestering specific *trans*-acting factors to prevent them from carrying out the native function in muscle.

Among the myopathies that arise from mutations in *cis*-regulatory elements, the most prevalent is Pompe disease where patients accumulate glycogen in their muscle, leading to hypotonia [155]. This accumulation of glycogen is a direct result of a T to G mutation (13T>G) in the intron 1 poly-pyrimidine tract of the *α-glucosidase* (*GAA*) pre-mRNA. This mutation reduces binding of the U2 component of the basal splicing machinery at the intron-exon boundary, causing partial skipping of exon 2 (containing the translation initiation codon) and reduced levels of cellular GAA [155]. These reduced levels of GAA cause a toxic buildup of glycogen in muscle lysosomes as the myofibers are unable to convert the complex sugar in its glucose energy source.

Myopathies can also arise from altered expression of *trans*-acting factors. Indeed, the decreased expression of the splicing factor SRSF1 has been shown to contribute to myositis through an altered selection of alternative 5′ splice sites on its target substrates [156]. In particular, the pro-inflammatory cytokine TNF-α has been shown to downregulate SRSF1 expression during myositis [156] thus contributing to modify the splicing profile of autoantigen transcripts, including *PM/Scl-100* and *PM/Scl-75* [157].

More often, muscular dystrophies are associated with the sequestration of *trans*-acting factors, preventing myofibers from establishing the full spectrum of muscle-specific splicing events needed for healthy tissue [15]. Myotonic dystrophy type 1 (DM1) and congenital myotonic dystrophy (CDM) are caused by expanded CUG repeats in the *DM protein kinase* (*DMPK1*) gene transcripts [158–161]. These expanded microsatellite repeats form a stable RNA secondary structure that sequesters the splicing regulator MBNL, which otherwise regulates the alternative splicing of multiple pre-mRNAs including *cardiac troponin* (*cTnT*) [162], *insulin receptor* (*INSR*) [163, 164], and muscle-specific *chloride channel* (*CLCN1*) [149, 165–169]. Perturbed splicing of *CLCN1* causes myotonia wherein defective muscle membrane chloride conductance causes hyperexcitability of myofibers resulting in repetitive action potentials [167]. A comprehensive study to understand *CLCN1* splicing in DM1 skeletal muscle tissue revealed the existence of aberrantly spliced *CLCN1* mRNAs with premature stop codons that arise due to retention of intron 2, inclusion of exon 6b and/or exon7a, and exclusion of exons 6 and 7 [167]. In the case of *INSR* splicing, DM1 is associated to the generation of the insulin signaling resistant isoform IR-A in the skeletal muscle cells thus reducing the metabolic effects of insulin on these cells [164, 170]. In addition to these perturbed splicing events, de novo sequestration of MBNL proteins causes a switch in alternative splicing that resembles embryonic alternative splicing patterns [171, 172]. This is illustrated by aberrant splicing of penultimate exon 78 of the *DMD* pre-mRNA, wherein sequestration or functional loss of MBNL1 results in the skipping of exon 78 [173]. Loss of exon 78 encodes for an embryonic version of dystrophin that causes abnormally ringed muscle fibers with disorganized Z band [173]. In another instance, aberrant alternative splicing of muscle-specific exon 11 of *bridging integrator*-1 (*BIN1*) generates an embryonic version of BIN1 (inactive) that lacks the phosphatidylinositol 5-phosphate-binding domain and generates disorganized T-tubule structures leading to perturbed calcium homeostasis, which is important for ECC in adult muscle fibers [174]. A shift in the alternative splicing pattern from adult to embryonic isoforms is also observed in a series of vesicular trafficking genes such synaptosome-associated protein 23 (*Snp23*), *thyroid hormone receptor interactor 10* (*Trip10)*, *clathrin heavy chain* (*Cltc*), and *transmembrane P24 trafficking protein 2* (*Tmed2*) leading to a significant disruption in T-tubule organization and mislocalization of the receptor proteins dihydropyridine receptor alpha (DHPR) and Ryr1 thus perturbing the release of calcium and causing myofibers enlargement and reduced force generation [175].

The sequestration of MBNL proteins is also observed in another form of myotonic dystrophy, DM2, where an expanded CCUG RNA repeat is encoded from intron 1 of *zinc finger protein 9* (*ZNF9*) [176]. DM1 is known to affect type I (slow) muscle fibers, while DM2 affects type II (fast) muscle fibers [177, 178]. Expanded RNA repeats in DM indirectly affect also additional RNA binding proteins. Sequestration of MBNL proteins alters nuclear signaling events, which eventually activate, via an unknown mechanism, the PKC pathway. Activated PKC hyperphosphorylates CUGBP1, another RNA binding protein, stabilizing it and facilitating its translocation to the nucleus where it regulates alternative splicing of multiple pre-mRNAs [179]. It has been reported that MBNL proteins and CUGBP1 antagonistically modulate the alternative splicing of several pre-mRNAs [89]. Transcriptome-wide binding studies revealed that nuclear MBNL proteins could either activate or repress specific splicing events. Binding of MBNL proteins upstream of the exon represses while binding downstream of the alternate exon enhances its inclusion [89, 180]. In the skeletal muscle, MBNL and CUGBP1 proteins regulate the alternative splicing of (at least) 120 exons among which 78 exons are regulated antagonistically [89, 180]. Staufen1 (Stau1), an RNA-binding protein that is known to regulate alternative splicing and translation efficiency, is increased in DM1 patients and mouse models [181]. Stau1 can also rescue *INSR* exon 11 alternative splicing in DM1 by binding to Alu elements in intron 10 thus acting as a modifier of DM1 disease severity [181, 182]. Thus, aberrant alternative splicing of multiple pre-mRNAs plays a key role to DM pathogenesis since it contributes to myotonia, muscle weakness, muscle wasting, insulin resistance, and defective calcium ion channels observed in DM patients.

Facioscapulohumeral muscular dystrophy (FSHD), another prevalent muscle disorder, involves copy number variation and/or epigenetic alteration of 3.3 kb tandem repeat sequences termed D4Z4 [183]. In healthy individuals, the FSHD locus is maintained in a repressed state, while the loss of silencing is observed in patients leading to aberrant expression of multiple nearby genes. Among this is the D4Z4 encoded double homeobox 4 (DUX4) transcription factor. Aberrant DUX4 activation in FSHD muscle disrupts RNA metabolism including pre-mRNA splicing [184–187]. Among DUX4 targets is *FSHD region gene 1* (*FRG1*), which has been found to co-purify with the C-complex of the spliceosome and regulate alternative splicing of multiple transcripts including *myotubularin-related protein 1* (*MTMR1*), and *troponin T type 3* (*TNNT 3*) pre-mRNAs in FSHD [183, 188, 189].

Muscular dystrophy can also be caused by *mis*-splicing of components of the dystrophin-glycoprotein complex (DGC) [190–192]. The DGC is a dynamic, multiprotein structure that connects the cytoskeleton to the extracellular matrix and plays important signaling and mechanical functions in the muscle [192]. DGC components include extracellular laminin-2, dystroglycan (α, extracellular; β transmembrane), sarcoglycan complex (α, β, γ, δ transmembrane glycoproteins), cytoplasmic dystrophin, dystrobrevins, and syntrophins [192]. The skeletal muscle cells express α- and γ-sarcoglycans, while the other family glycoproteins are widely expressed across all cell types [192, 193]. However, in rats, alternative splicing of *δ-sarcoglycan* yields a skeletal muscle-specific isoform termed as δ-SG3 (devoid of 122 aa in the C-term) that was shown to maintain the calcium homeostasis in the sarcoplasmic reticulum [194].

On the cytoplasmic side of the DGC, we have the syntropins and dystrobrevin complex proteins [192]. Dystrobrevin (DB) exists as α-DB and β-DB. *α-DB* undergoes alternative splicing to generate five different isoforms, among which *α-DB1* and *α-DB2* are predominant and are expressed exclusively in the muscle cells [195, 196]. These splice variants differ in their C-terminus and exhibit differential localization, such as α-DB2 is localized to sarcolemma of neuromuscular junction and co-purifies with dystrophin [195, 197, 198]. On the contrary, α-DB1 associates with utrophin and dystrophin with a better affinity for utrophin and localizes to the synapse. These α-DBs are indispensable for signaling in the muscle and neuromuscular synaptogenesis. However, in DM1 patients, splicing of *α-DB1* is dysregulated and results in the enhanced inclusion of exons 11A and 12, encoding for variable region 3 [198]. The inclusion of variable region 3 enhances α-DB1 association with α-syntrophin thus leading to altered phosphotyrosine signaling at neuromuscular junction [198].

**Fig. 6:**
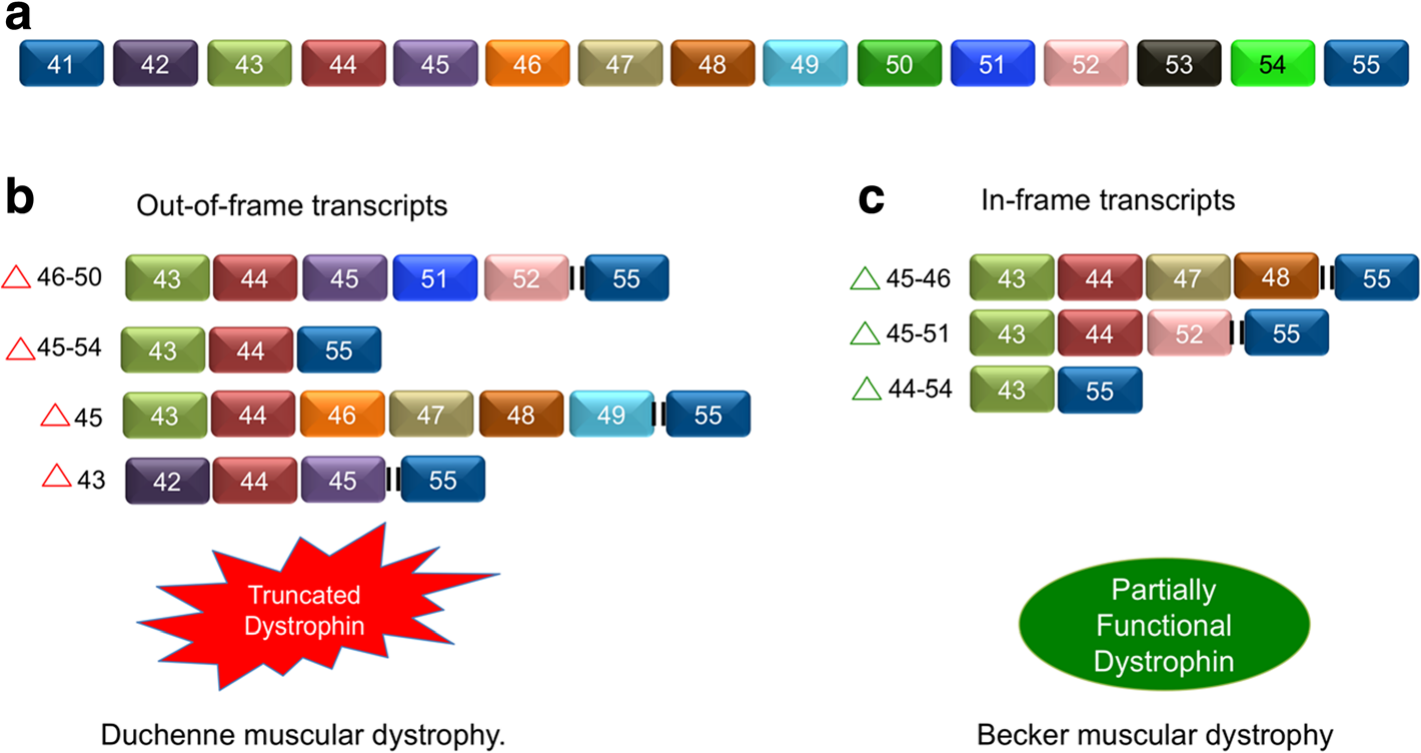
Alternative splicing in Duchenne muscular dystrophy. **a** Genomic organization of *dystrophin* gene locus. **b** Aberrant alternative splicing results in the formation of “out-of-frame” transcripts that cannot encode for functional dystrophin, causing Duchenne muscular dystrophy (DMD). **c** However, some of the splicing events also yield “in-frame” transcripts that can encode for partially functional dystrophin, the phenotype of which is called Beckers’ syndrome or Becker muscular dystrophy (BMD)

Aberrant splicing of a single exon (43, 45) or multiple exons (46–50; 45–54) in *dystrophin* pre-mRNA results in the generation of stop codons in exon 44, 51, or 55, and as a consequence, non-functional protein products [199]. Deletion of both the exons (45, 46) and (45, 51) or multiple exons (45–51) can restore the reading frame, resulting in the formation of a partially functional dystrophin protein that is associated with a milder form of the disease called Becker muscular dystrophy (BMD) (Fig. 6) [199, 200].

Interestingly, a patient with an exon 44 deletion in the *dystrophin* gene that would be expected to lead to DMD was found to present with a BMD phenotype [201]. Characterization of this patient showed that expression of the splicing factor CELF2a (CUGBP, Elav-like family member 2) was downregulated in the muscle through an unknown mechanism. The loss of CELF2a acts as disease modifier by reducing the inclusion of exon 45 in the *dystrophin* mRNA, re-establishing the open-reading frame, and resulting in the formation of a partially functional dystrophin protein [201].

The reduced myopathy through exon skipping observed in the above DMD patient suggests that controlling alternative splicing of *dystrophin* pre-mRNA could have therapeutic value. Consistent with such an idea, the drug eteplirsen was developed to specifically induce exon 51 skipping in the *dystrophin* mRNA and has recently been approved by the FDA for the treatment of DMD [202]. Similarly, trials are underway to test anti-sense oligonucleotides (AON) to induce alternative exon usage for the therapy of other muscle diseases. For example, AON therapy targeted against the 3′ splice site of exon 7a of *CLCN1* pre-mRNA was found to effectively eliminate chloride channelopathy in DM1 mouse models by inhibiting the inclusion of exon 7a, which restores the full-length reading frame [203]. Using a more general approach, a 25-mer CAG repeat morpholino oligos that binds CUG^exp^, injected into the muscle fibers of a DM1 mouse, can effectively displace the sequestered MBNL proteins from the CUG repeat RNA and restore the majority of the MBNL/CELF dependent splicing events, reversing the manifestations of myotonic dystrophy [204].

The success observed in generating these highly specific therapeutics underscores the importance of understanding the mechanisms of alternative splicing regulation for the development of novel treatment for myopathies.

## Perspectives and future directions

Exploring novel function for proteins in an unbiased manner often starts with a “guilt by association” approach that probes for factors that co-immunoprecipitate in muscle environments. Identifying several peptides by mass-spectrometry, proteins not previously known to interact with the factor of interest provide new functional insight based on a well-defined role for the interactor in the existing muscle literature. However, the peptides identified by mass spectrometry for these novel interactors often lack sufficient protein coverage to provide information on the specific splice isoform involved in the interaction. In the absence of an isoform-specific antibody, it is difficult to know with which splice variant your protein of interest might interact. Therefore, there is the potential for obtaining false negative results when performing reciprocal immunoprecipitations in confirmation studies if the antibody does not recognize all forms of the interacting protein. The emergence of new targeted proteomic technologies such as selective reaction monitoring (SRM) allows us to begin getting around this problem as it enables for the characterization and quantification of specific peptides using mass spectrometry and thus has the potential to identify the specific protein isoforms [205]. By telling the mass spectrometer to scan tryptic peptides that would be specific to distinct splicing isoforms of your protein of interest, it is now becoming possible to refine the interaction networks where specific protein isoforms can be defined. Application of this technology to alternative spliced isoforms has the potential to provide us with a new layer of understanding with respect to the muscle proteome and its related interactome. That being said, one must be wary of the large number of alternative splicing events that are identified in our genome-wide studies. While algorithms used to identify alternative transcripts might deem a splicing event statistically significant, this does not imply that the splicing event has biological significance. Indeed, many of the computationally defined alternative splicing events give rise to only a tiny fraction of the total transcripts for a gene in the cell. As such, it is unclear which alternatively spliced transcripts are contributing abundantly to the total amount of the encoded protein, and whether these alternative isoforms contribute to an altered muscle function. Thus, when embarking on a project to characterize an annotated muscle-specific splice variant, it would be prudent to first confirm that this splice variant represents a major isoform in muscle cells.

In terms of regulating splicing events, we continue to have an incomplete understanding of the mechanism that direct alternative exon usage to generate distinct protein isoforms in muscle. Mining of high-throughput data has led to the elucidation of many tissue-specific *cis*-regulatory elements and *trans*-acting splicing factors responsible for modulating alternative splicing in the muscle cells [41, 78]. However, we have very little insight into the role played by signal transduction pathways that could temporally and spatially regulate the function of *trans*-acting splicing factors thus permitting dynamic adjustments in the muscle proteome in response to external stimuli. Signaling transduction such Wnt, Notch, TGFβ, and MAPK have been extensively studied for their role in regulating gene expression during muscle development and function [206–210]. However, we have not yet begun exploring how these signaling pathways influence the splicing machinery and thus alter the splicing decisions. Looking at splicing in different cellular contexts, there is certainly reason to believe that these signaling pathways may contribute to the regulation of alternative splicing in the muscle. Studies in cancer cells have shown that Wnt-mediated activation of β-catenin regulates alternative splicing of the *estrogen receptor β* pre-mRNA through the upregulation of the *trans*-activating splicing factor SRSF3 [211, 212]. TGF-β signaling stimulates an interaction between SMAD3 and the *trans*-acting splicing factor PCBP1, targeting the splicing machinery to the *CD44* pre-mRNA to affect alternative exon usage [213]. Similarly, activation of the p38 MAPK signaling pathway controls localization and or post-translational modifications of various trans-acting factors including Sam68, hnRNPA1, etc., causing them to accumulate in the cytoplasm and affecting, as a consequence, alternate splicing of *CD44* pre-mRNA, adenovirus *E1A* pre-mRNA, etc. [214, 215]. Sam68, a member of the signal transduction and activation of RNA family of RNA binding proteins, is an important regulator of *SMN2* splicing in spinal muscular atrophy [216]. These diverse examples of signal-induced differential splicing events highlight the potential for alternative splicing to play a relevant role in defining the cellular response to environmental cues and thus underscore the necessity to delineate the mechanisms and events of alternative splicing regulation in muscle in response to various signaling pathways.

Beyond the direct regulation of the splicing machinery by signaling pathways, recent studies have suggested that epigenetic modification of histones and DNA can also contribute to the regulation of alternative splicing [26, 27]. Studies by Luco et al. [26] demonstrated that histone modifications and or chromatin modifiers such as H3K36 methyltransferase SETD2 can regulate the inclusion of alternative exons usually repressed by PTB but not of PTB-independent exons [26]. Considering that PTB binds to conserved *cis*-regulatory elements “CUCUCU” and influences muscle cell-specific alternative splicing events, it will be interesting to delineate how various histone modifications and other chromatin regulatory factors would influence tissue-specific alternative splicing during muscle-cell differentiation [27, 31, 217]. Thus, combining these perspectives with high-throughput systematic global analyses will be a great resource to understand muscle-specific alternative splicing events and thus elucidate muscle-associated diseases.

## Conclusions

Modulation of alternative splicing during development represents a major mechanism through which muscle cells diversify their functional proteome. The majority of the studies to date have delineated alternative splicing patterns during differentiation, regulated by cell type-specific *trans*-acting factors that bind to *cis*-regulatory motifs and dictate either the inclusion or skipping of the specific alternative exon. However, there is a need to link the modes of alternative splicing with signaling cues that play a pivotal role during embryonic myogenesis as well as during adult muscle regeneration. With the advent of high-throughput technologies, it is important to combine genomic and proteomic approaches to identify the unique targets whose alternative splicing is regulated in a signal-dependent manner during myogenesis. Muscle SCs represent an ideal model system for delineating the signal-dependent and signal-independent alternative splicing mechanisms during differentiation. Future studies in this direction will be valuable for understanding the complexity of muscle biology while unveiling additional levels at which alternative splicing contributes to the pathology of different myopathies.

## Additional file

Additional file 1: List of genes that undergo alternative splicing in muscle tissue [218–226]. (DOCX 36 kb)

## Abbreviations

BMD: Becker muscular dystrophy
CELF: CUG-binding protein Elav-like family member
CLIP: Cross-linking immunoprecipitation
CTD: Carboxyl terminal domain
DM1 & DM2: Myotonic dystrophy type 1 & 2
DMD: Duchenne muscular dystrophy
HDAC: Histone deacetylase
INSR: Insulin receptor
MAPK: Mitogen-activated protein kinases
MBNL: Muscleblind-like protein
MEF2: Myocyte enhancer factor-2
Myf5: Myogenic factor 5
MyoD: Myoblast determination protein
PKA: Protein kinase A
PTB: Polypyrimidine tract-binding protein
RBM: RNA-binding motif
SCs: Satellite cells
SF1: Splicing factor 1
snRNPs: Small nuclear ribonucleoproteins
