# Supplementary material for: Diversification of the muscle proteome through alternative splicing
Source: Skelet Muscle. 2018 Mar 6;8:8. doi: 10.1186/s13395-018-0152-3 (PMC5840707; doi:10.1186/s13395-018-0152-3)
Supplement: Supplementary file 1 — List of genes that undergo alternative splicing in muscle tissue [218–226]. (DOCX 36 kb) [file 13395_2018_152_MOESM1_ESM.docx]

**Additional file 1: Table S1: List of genes that are differentially spliced during myogenesis**

| **Gene Name [Ref]** | **Gene Description** | **Exon^(Change)^** | **Trans-factor** | **Functional Significance** |
| --- | --- | --- | --- | --- |
| BIN1 [174,218] | Bridging Integrator 1 | 11^(In)^ | MBNL | Adult form; Biogenesis of muscle T tubules |
|  |  | 11^(Ex)^ | CELF | Fetal form; Defective T tubule; Perturbed Ca^2+^ homeostasis; Altered excitation-contraction coupling. |
| CAPZB [87] | Capping Actin Protein Of Muscle Z-Line Beta Subunit | 9^(In)^ | Inclusion by QK1; Exclusion by PTB; SRSF10 | Assembly and stabilization of actin filaments. Muscle cell specific isoform. |
| Ca_v_1.1/CACNA1S [138,219] | Calcium Voltage-Gated Channel Subunit Alpha1 S | 29^(In)^ |  | Voltage gated Ca^2+^ channel; Conducts a slowly activating Ca^2+^ current |
|  |  | 29^(Ex)^ | MBNL/RBFOX2 | Open channel; Accelerated Ca^2+^ currents; |
| Ca_v_1.2/CACNA1C [140] | Calcium Voltage-Gated Channel Subunit Alpha1 C | 9^(In)^; 33^(Ex)^ | RBFOX proteins ↓ | Hyperpolarized Ca^2+^ shifts for inactivation and activation potentials |
|  |  | 9^(Ex)^; 33^(In)^ | RBFOX proteins ↑ |  |
| CLCN1 [167,168] | Chloride Voltage-Gated Channel 1 | Retention of Intron 2  6b, 7a | MBNL, CELF | Premature termination codons target splice variants for mRNA decay; Loss of chloride ion channel |
| Cltc [175] | Clathrin Heavy Chain | 21nt^(Ex)^ |  | Embryonic; Less efficient endocytosis and actin scaffolding; Defective T-tubule Organization |
|  |  | 21nt^(In)^ |  | Adult form; functional and efficient in actin scaffolding and T-tubule organization |
| CNNM3 [220] | Cyclin and CBS Domain Divalent Metal Cation Transport Mediator 3 | 2^(Ex)^ |  | Loss of magnesium transport required for muscle contraction |
| DEPDC1 [220] | DEP Domain-Containing Protein 1A | 8 |  | Transcriptional regulation, mitotic progression, albeit not reported in the context of myogenesis |
| DMD [199,200] | Dystrophin | 43^(Ex)^, 45^(Ex)^;  45-54^(Ex)^; 46-50^(Ex)^ |  | Muscle integrity |
| Fxr1 [98,221–223] | Fragile X Mental Retardation Syndrome-Related Protein 1 | 15^(In)^, 16^(In)^ | SRSF10 | Muscle cell specific inclusion, Binds p21 mRNA and reduces half-life, regulating cell cycle exit |
| Gpx8 [224] | Glutathione Peroxidase 8 | 2^(In)^ |  | Exon included in adult; lost in diabetic mice |
| IFRD1 [220] | Interferon Related Developmental Regulator 1 | 5^(In)^ |  | Transcriptional regulator; excluded in cachexia |
| INSR [163,164] | Insulin Receptor | 11^(In)^ | MBNL | Sensitive to insulin, higher signaling potential; Adult form |
|  |  | 11^(Ex)^ | MBNL/CELF | Insulin insensitive form/ Insulin resistance; fetal form. |
| KCNQ5 [220] | Potassium Voltage-Gated Channel Subfamily Q Member 5 | 9^(In)^ |  | Potassium voltage-gated channel; Exon lost in cachexia |
| Lrrfip1 [98,225] | LRR Binding FLII Interacting Protein 1 | 16^(In)^,17^(In)^ | SRSF10 | Strongest enhancer of wnt/β-catenin canonical transcription pathway during myogenesis |
| MEF2A [98] | Myocyte Enhancer Factor 2A | 9^(In)^ | SRSF10 | Unknown effect on gene expression |
| MEF2C [59–61] | Myocyte Enhancer Factor 2C | α1^(ME)^ |  | α1 associates with HDAC4 & 5 but not α2; |
|  |  | α2^(ME)^ |  | α2 does not associate with HDAC4 & 5 |
|  |  | γ^(In)^ |  | γ possess phosphorylation site |
| MEF2D [64,66] | Myocyte Enhancer Factor 2D | α1^(ME)^ | RBFOX1/2 | Transcriptional repressor; α1 associates with HDAC4 and 9 |
|  |  | α2^(ME)^ |  | Transcriptional activator; Associates with Ash2L but not with HDACs |
| MICU1 [132] | Mitochondrial Calcium Uptake 1 | μ exon 4^(In)^ |  | Efficient binding of mitochondrial Ca^2+^ |
| Mtmr1 [226] | Myotubularin Related Protein 1 | 2.1, 2.2, 2.3^(In)^ | CUGBP1 | Phosphoinositide phosphatase |
| Mtmr3 [224] | Myotubularin Related Protein 3 | 16^(In)^ |  | Exon included in adult |
|  |  | 16^(Ex)^ |  | Exon excluded in fetal form; lost in diabetic mice |
| Ppp3ca [44,224] | Protein Phosphatase 3 Catalytic Subunit Alpha | 13^(In)^ |  | Affects auto-inhibitory domain. Regulates Nfatc3 activity |
| Ppp3cb [44] | Protein Phosphatase 3 Catalytic Subunit Beta | 10a^(In)^; 13^(In)^ |  | 10a, affects calmodulin domain; 13, affects auto-inhibitory domain; Regulates Nfatc3 activity |
| Ppp3cc [44] | Protein Phosphatase 3 Catalytic Subunit Gamma | 10a^(In)^; 13^(In)^ |  | 10a, affects calmodulin domain; 13, affects auto-inhibitory domain; Regulates Nfatc3 activity |
| RyR1 [136,146] | Ryanodine Receptor 1 | 70^(Ex)^, 83^(Ex)^ | RBFOX1/CUGBP1 | Impaired Ca^2+^ Homeostasis; impaired excitation-contraction coupling |
| SERCA1 [150,152,154] | Sarcoplasmic/Endoplasmic Reticulum Calcium ATPase 1 | 22^(In)^ | RBFOX1 | SERCA1a, Adult form; Fast-twitch fibers; Higher ATPase and Ca^2+^ handling activity |
|  |  | 22^(Ex)^ |  | SERCA1b, Fetal form; Lower ATPase and Ca^2+^ handling activity |
| SERCA2 [146,151,152] | Sarcoplasmic/Endoplasmic Reticulum Calcium ATPase 2 | 21^(In)^ |  | Slow-twitch fibers; Altered ATPase and Ca^2+^ handling activity |
|  |  | Intron 19 retention |  | Expressed in normal muscle cells. Downregulated in DM1pathogenesis |
| SNAP23 [175] | Synaptosome Associated Protein 23 | 6^(In)^, 33nt |  | Adult form, facilitates glucose metabolism and secretion |
|  |  | 6^(Ex)^ |  | Fetal and early post-natal form; Insulin resistance in diabetic skeletal muscle |
| Tmed2 [175] | Transmembrane P24 Trafficking Protein 2 | 21nt^(In)^ |  | Adult form, facilitates cargo selection in secretion; 21nt exclusion in fetal form of Tmed2 |
| TRIP10 [175] | Thyroid Hormone Receptor Interactor 10 | 168nt^(In)^ |  | Required for membrane deformation, tubulation and vesicular trafficking |
| Tropomyosin [114,115,117,126] | Tropomyosin 1 | 2^(In)^ |  | Smooth muscle specific; Alters affinity with actin and regulates actin assembly; Muscle contraction |
|  |  | 3^(In)^ |  | Skeletal muscle specific; Differential affinity with F-actin |
|  |  | 5- NM; SK |  | Exon 5 NM, inclusion in non-muscle cells; exon 5 SK, inclusion in skeletal muscle cells. |
| Troponin [120,162] |  | 16/17^(ME)^, 4 ^In/Ex^, 5 ^In/Ex^ |  | Cell-type specific inclusion/exclusion; Muscle contraction |
| UBA3 [220] | Ubiquitin Like Modifier Activating Enzyme 3 | 4 ^(Ex)^ |  | Protein ubiquitination |
| α-Dystrobrevin [195–197] | α− Dystrobrevin1,  α− Dystrobrevin2 | 11A^(Ex)^ 12^(Ex)^ |  | Skeletal muscle specific isoforms. |
|  | α− Dystrobrevin1 | Y-Tyrosine kinase substrate domain ^(IN)^ |  | Associates with dystrophin, utrophin; highly restricted to synapse |
|  | α− Dystrobrevin2 | Y-Tyrosine kinase substrate domain^(Ex)^ |  | Associates with dystrophin only, no interaction with Utrophin; associated with sarcolemma |
| δ-sarcoglycan [194] | Sarcoglycan Delta | 9^(Ex)^ |  | Tissue specific, maintains Ca^2+^ homeostasis |

^(Ex)^ Exon Exclusion; ^(In)^ Exon Inclusion; ^(ME)^ Mutually Exclusive; ↑Upregulated; ↓Downregulated
